# High-Resolution Forward-Looking Imaging Method for FMCW Radar Based on Sparse Sampling

**DOI:** 10.3390/s26165016

**Published:** 2026-08-07

**Authors:** Qin Zhao, Xiaopeng Yan, Tao Zhang, Qingyu Hou, Qiang Liu, Jiawei Wang, Xinwei Wang

**Affiliations:** 1 National Key Laboratory of Proximity Detection and Control, Beijing Institute of Technology, Beijing 100081, China; 3120225131@bit.edu.cn (Q.Z.);; 2 Nanjing Institute of Electronic Equipment, Nanjing 211103, China; 3 China Academy of Ordnance Science, Beijing 100089, China

**Keywords:** FMCW radar, forward-looking imaging, adaptive beamforming, Doppler ambiguity, grating lobes, sparse sampling intervals

## Abstract

Platform-induced synthetic aperture is an effective approach to enhancing azimuth resolution in forward-looking radar imaging. However, for small platforms such as automobiles and unmanned aerial vehicles, the large volume of echo data required under continuous sampling, combined with the presence of Doppler ambiguity, poses substantial challenges for high-resolution imaging. To address these issues, this paper proposes a forward-looking FMCW radar imaging method based on sparse sampling intervals. A uniform linear array is first employed to acquire measurements at different platform positions, and an initial range-angle image is obtained for each channel. Adaptive beamforming is then applied to impose nulls on false-alarm regions, including grating lobes and left-right ambiguity. Finally, coherent accumulation across channels yields a high-resolution range-angle image. Simulation and experimental results demonstrate that the proposed method achieves high-resolution forward-looking imaging while significantly reducing the volume of echo data.

## 1. Introduction

In recent years, forward-looking radar imaging has demonstrated significant application potential in military surveillance, autonomous driving, and medical devices [1,2,3,4]. Key challenges in these scenarios include improving angular resolution [5,6], resolving Doppler ambiguities [7], and developing fast imaging algorithms suitable for small platforms [8].

Angular resolution is fundamentally determined by the antenna aperture. A common approach is to deploy an array antenna at the front end of the platform and employ spatial spectrum estimation techniques—such as conventional beamforming (CBF) [9,10], multiple signal classification (MUSIC) [11,12], estimation of signal parameters via rotational invariance techniques (ESPRIT) [13,14], and maximum likelihood (ML) estimation [15,16]—to obtain high-resolution angle estimates. However, for compact platforms such as automobiles and unmanned aerial vehicles, physical space constraints prevent the use of large-aperture arrays, resulting in limited angular resolution improvement. To overcome this limitation, scholars have explored optimized array configurations such as minimum redundancy arrays [17,18], nested arrays [19,20], and coprime arrays [21,22], as well as MIMO array techniques [23,24]. Although these approaches achieve larger virtual apertures and higher degrees of freedom using fewer antenna elements, their resolution improvement remains constrained by platform size, and the array structures themselves tend to be complex and costly. Synthetic aperture radar (SAR) [25,26] and Doppler beam sharpening (DBS) [27,28] represent another class of methods that effectively enhance angular resolution by exploiting platform motion to synthesize a large virtual aperture. While both techniques exhibit blind zones in forward-looking configurations, they significantly improve angular resolution outside the blind area and can reduce the extent of the blind zone [29].

This paper focuses on several fundamental challenges associated with forward-looking imaging. The first is Doppler ambiguity: targets symmetric with respect to the platform trajectory exhibit identical Doppler signatures, producing symmetric false angles in the range-angle domain. Various solutions have been proposed. For example, ref. [30] employs bistatic SAR using an additional transmitter placed at a different location to acquire supplementary information and eliminate ambiguity. A multichannel radar system in [31] combines back-projection (BP) imaging with a spatial null-constrained adaptive beamforming method, though at the expense of high computational complexity. A scanning imaging system in [32] resolves ambiguity through multi-beam DBS, while ref. [7] multiplies the angular profiles obtained from DBS and real-array DBF processing to suppress ambiguity; however, this approach widens the beamwidth and degrades the ability to resolve closely spaced targets.

Another major challenge arises from the large volume of echo data generated by continuous sampling during platform motion, which imposes a substantial computational burden on resource-limited small platforms and severely affects real-time imaging performance. To reduce data volume, compressed sensing theory [33,34] has been applied to exploit signal sparsity and reconstruct images from undersampled measurements, though reconstruction quality heavily depends on the sensing matrix. In [35], density weighting and simulated annealing are used to optimize the azimuth aperture, and a non-uniform Taylor window with zero filling is applied to achieve SAR imaging with an optimized sparse aperture. The work in [36] analyzes aliasing effects caused by sparse sampling in both the wavenumber and spatial domains and proposes a Fourier-based sparse MIMO-SAR imaging method, albeit with high computational complexity.

Motivated by the need for high angular resolution, ambiguity suppression, and fast imaging (i.e., low data volume) in forward-looking radar scenarios, this paper proposes a high-resolution imaging method based on sparsely spaced sampling intervals. Platform forward motion is exploited to perform sparse, uniformly spaced sampling in the slow-time domain. After analyzing the effects of sparse sampling and Doppler ambiguity, an optimized beamforming strategy is employed to suppress grating lobes and ambiguity, enabling the reconstruction of a high-resolution range-angle image. Unlike traditional sparse-aperture SAR/MIMO-SAR methods that often rely on random or non-uniform sub-sampling and complex sparse reconstruction algorithms (e.g., compressed sensing with iterative optimization), our method adopts uniform sparse sampling intervals in the slow-time domain. This design allows us to adopt an FFT-based processing framework rather than relying on iterative convex optimization. Consequently, our approach achieves computational efficiency while the sparse interval significantly reduces data volume. Compared with prior multi-channel forward-looking SAR methods, we utilize the physical array not just for high-resolution imaging but to generate explicit nulls at the theoretically predicted false-target positions (both mirror ambiguities and grating lobes) using the LCMV criterion. This significantly reduces computational complexity because we bypass the heavy iterative back-projection used in [31].

The main contributions of this work are summarized as follows:

A sparse slow-time sampling signal model is established, which significantly reduces the required data volume while enabling high-angular-resolution imaging.

The impact of sparse sampling on imaging performance is analyzed, and an adaptive beamforming-based optimization algorithm is proposed to suppress grating lobes and left-right ambiguities, yielding high-resolution and ambiguity-free images.

The remainder of this paper is organized as follows. Section 2 analyzes the forward-looking radar geometry, establishes the signal model, and discusses error sources in FMCW radar imaging. Section 3 presents the problem analysis and the proposed algorithm. Section 4 provides simulation and experimental validation. Finally, Section 5 concludes the paper.

## 2. Imaging Geometry and Signal Model

### 2.1. Forward-Looking Radar Imaging Geometry

**Figure 1:**
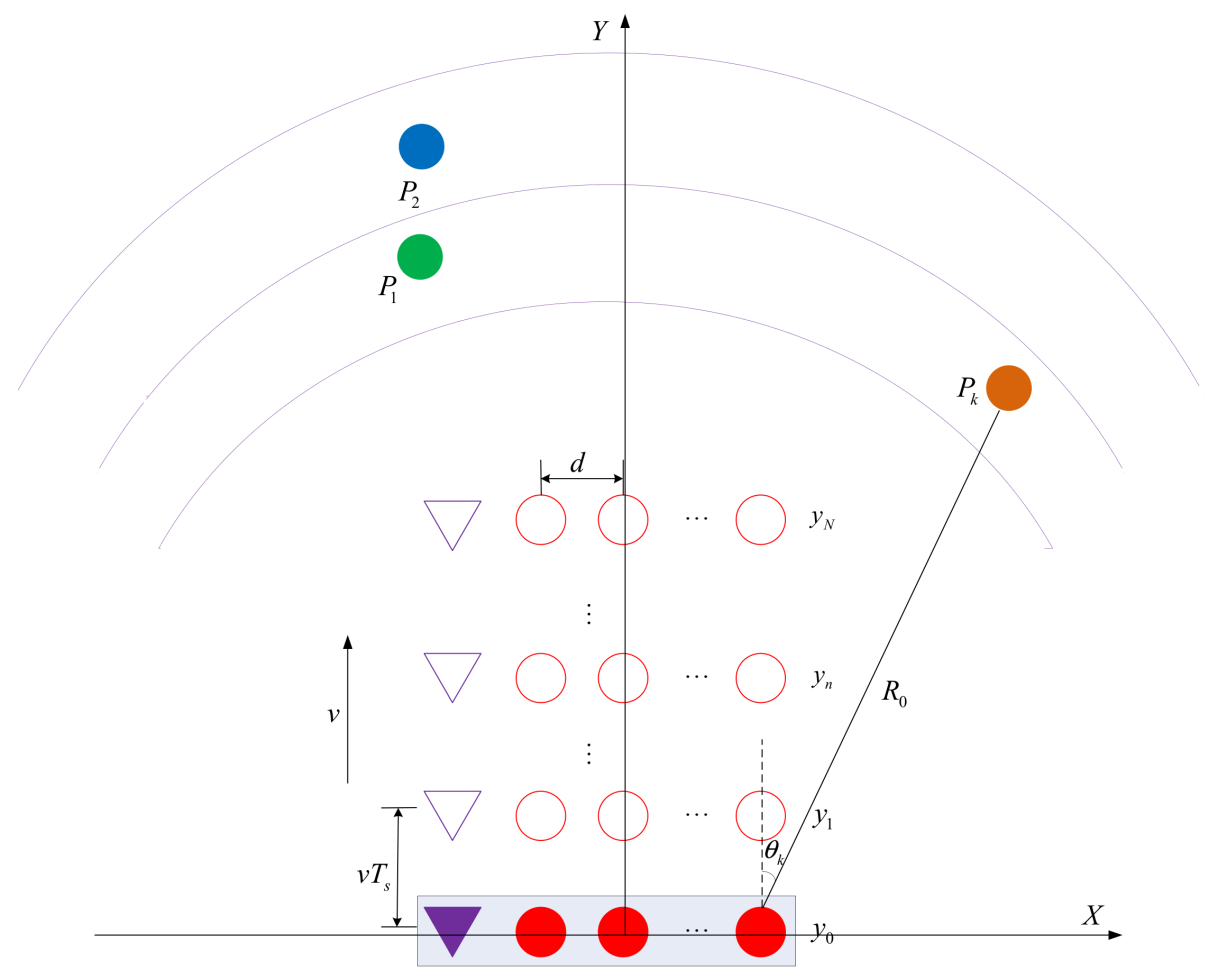
Forward-looking radar imaging geometry.

The forward-looking imaging geometry is illustrated in Figure 1. A uniform linear array (ULA) with a single transmitter and multiple receivers is deployed along the x-axis. The platform moves at a constant velocity v along the array’s normal direction, i.e., the y-axis. The purple triangle represents the transmit antenna, and the red circles denote the M receive elements with inter-element spacing *d*. The radar positions are denoted as $y_{0}$ for the initial location, $y_{n}$ for the *n*-th sampling position, and $y_{N}$ for the position after the platform has completed its motion.

Targets $P_{1}$, $P_{2}$, $P_{k}$ are located on both sides of the y-axis. The angle $\theta_{k}$ represents the direction of the target $P_{k}$ with respect to the ULA broadside. The initial range from the radar to a target is denoted by $R_{0}$, and $T_{s}$ denotes the slow-time sampling interval.

When only the physical array is used for forward-looking imaging, the range resolution and angular resolution are given by (1) and (2), respectively.

(1) $$\rho_{r}=\frac{c}{2B}$$

(2) $$\theta_{res}\approx 0.886\frac{\lambda }{D_{real}}=0.886\frac{\lambda }{\left(M-1\right)d}$$

where c denotes the speed of light, B is the signal bandwidth, λ represents the wavelength, and D is the physical array aperture. It follows that the ideal range resolution can be improved by increasing the transmit bandwidth, whereas the angular resolution is determined by the physical aperture. When the aperture is limited, achieving sufficiently high angular resolution becomes difficult, making it impossible to distinguish closely spaced targets such as $P_{1}$ and $P_{2}$ in Figure 1.

### 2.2. Signal Model

The scenario considered in this paper satisfies the far-field and narrowband assumptions. The transmitted signal is an FMCW waveform, expressed within a single chirp period as

(3) $$s_{t}(\hat{t})=\exp(j2\pi (f_{0}(\hat{t})+\frac{1}{2}\beta {\hat{t}}^{2})),nT\leq \hat{t}\leq (n+1)T$$

where $f_{0}$ is the carrier frequency, *T* is the chirp duration, β=B/T is the frequency modulation slope, and $\hat{t}$ denotes the fast time within a single chirp period, n=0,1,⋯N−1. The parameter *N* represents the number of chirp periods, i.e., the total number of slow-time samples. After reflection from the target, the echo received by the *m*-th array element can be written as

(4) $$s_{r}(t,m)=\sum_{k=1}^{K}\exp(j2\pi (f_{0}(t-\tau \left(m,k\right))+\frac{1}{2}\beta {\left(t-\tau \left(m,k\right)\right)}^{2}))$$

where *K* denotes the total number of targets, and $\tau \left(m,k\right)$ represents the round-trip propagation delay between the *m*-th receive element and the *k*-th target, given by

(5) $$\tau \left(m,k\right)=(R_{k}(t,m)+R_{k}(t,s))/c$$

where *t* denotes the continuous time, $t=t_{n}+\hat{t}$, $t_{n}$ is the slow time, $t_{n}=nT,n=0,1,\cdots N-1$. $R_{k}(t,m)$ represents the instantaneous slant range between the *m*-th receive element and the target, while $R_{k}(t,s)$ denotes the instantaneous slant range between the transmit element and the target, which are expressed as

(6) $$R_{k}(t,m)=\sqrt{R_{0}^{2}\left(k,m\right)+{\left(vt\right)}^{2}-2R_{0}\left(k,m\right)vt\cos\theta_{k}}+dm\sin\theta_{k}$$

(7) $$R_{k}(t,s)=\sqrt{R_{0}^{2}\left(k,s\right)+{\left(vt\right)}^{2}-2R_{0}\left(k,s\right)vt\cos\theta_{k}}$$

where $R_{0}\left(k,m\right)$ is the initial range between the *m*-th receive element and the target, $R_{0}\left(k,s\right)$ is the initial range between the transmit element and the target, and $\theta_{k}$ denotes the angle of the *k*-th target.

In the scenario considered in this paper, the array is compactly configured, and the difference between $R_{0}\left(k,m\right)$ and $R_{0}\left(k,s\right)$ is much smaller than the radar’s range resolution. Thus, they can be approximated as equal, yielding.

After mixing the echo received by the *m*-th element with the reference signal, the resulting beat signal is given by

(8) $$s_{beat}(t,m)=\sum_{k=1}^{K}\exp(j\frac{4\pi }{c}(f_{0}+\beta \hat{t})R_{k}(t,s))\exp(-j\frac{2\pi }{c}(f_{0}+\beta \hat{t})dm\sin\theta_{k})\cdot \exp(j\frac{4\pi \beta R_{k}^{2}(t,s)}{c^{2}})$$

Unlike pulse-based radar imaging, FMCW imaging introduces additional error sources that must be considered. This section provides an in-depth analysis of these effects.

By neglecting third- and higher-order terms and performing a Taylor expansion of $R_{k}(t,s)$ around the fast time $\hat{t}=0$, we obtain

(9) $$R_{k}(t,s)=R_{n}+k_{n}\hat{t}$$

where $R_{n}$ denotes the instantaneous slant range under the stop-go-stop model, and $k_{n}$ represents the error induced by the platform’s continuous motion during a single chirp period.

(10) $$\begin{array}{l} R_{n}=\sqrt{R_{0}^{2}\left(k,s\right)+{\left(vt_{n}\right)}^{2}-2R_{0}\left(k,s\right)vt_{n}\cos\theta_{k}}\\ k_{n}=\frac{v^{2}t_{n}-R_{0}\left(k,s\right)v\cos\theta }{\sqrt{R_{0}^{2}\left(k,s\right)+{\left(vt_{n}\right)}^{2}-2R_{0}\left(k,s\right)vt_{n}\cos\theta_{k}}} \end{array}$$

Thus, (8) can be rewritten as

(11) $$\begin{array}{ll} s_{beat}(t,n,m) & =\sum_{k=1}^{K}\exp(j\frac{4\pi }{c}(f_{0}+\beta \hat{t})R_{n})\exp(-j\frac{2\pi }{c}(f_{0}+\beta \hat{t})dm\sin\theta_{k})\\ & \cdot \exp(j\frac{4\pi }{c}f_{0}k_{n}\hat{t})exp(j\frac{4\pi }{c}\beta k_{n}{\hat{t}}^{2})\exp(j\frac{4\pi \beta R_{k}^{2}(t,s)}{c^{2}}) \end{array}$$

where the third exponential term corresponds to the range shift error. In the fourth exponential term, since $\beta k_{n}{\hat{t}}^{2}\leq Bk_{n}\hat{t}\ll f_{0}k_{n}\hat{t}$, its influence on the result is much smaller than that of the third term, and can therefore be neglected. The fifth exponential term represents the residual video phase introduced by the beat-frequency processing, which can be removed through range-domain matched filtering.

The frequency error introduced by the third exponential term is given by

(12) $$\Delta f=\frac{2}{c}f_{0}k_{n}$$

The corresponding range error in the range dimension is

(13) $$\Delta R=\frac{c\Delta f}{2\beta }=\frac{ck_{n}}{\lambda \beta }=\frac{cv}{\lambda \beta }\cos\theta =\frac{c}{2\beta }f_{d}$$

where $f_{d}$ denotes the Doppler frequency. Defining Δp as the ratio between the range shift error and the range resolution, we obtain

(14) $$\Delta p=\frac{\Delta R}{\rho_{r}}=\frac{cf_{d}}{2\beta }\cdot \frac{2B}{c}=\frac{f_{d}}{f_{m}}$$

where $f_{m}$ denotes the modulation frequency of the signal. When Δp>0.5, defocusing occurs in the range dimension. For FMCW radar platforms operating at high carrier frequencies and high velocities, the Doppler frequency is typically large, and the resulting range shift error may become non-negligible. In such cases, compensation must be incorporated into the imaging process.

By performing a Taylor expansion of $R_{n}$ in the first exponential term and neglecting higher-order components, we obtain

(15) $$R_{n}=R_{0}\left(k,s\right)-vt_{n}\cos\theta_{k}+\frac{v^{2}\sin^{2}\theta_{k}}{2R_{0}\left(k,s\right)}t_{n}^{2}$$

The second term represents range walk, while the third term corresponds to range curvature. In forward-looking scenarios, the target angle $\theta_{k}$ is typically small compared with side-looking configurations, making the impact of range walk significantly greater than that of range curvature. When extremely high accuracy is not required, the effect of range curvature can therefore be neglected.

After applying the above compensation and approximations, the expression of the forward-looking beat signal can be written as follows:

(16) $$\begin{array}{ll} s_{beat}(t,n,m) & =\sum_{k=1}^{K}\exp(j\frac{4\pi }{c}(f_{0}+\beta \hat{t})R_{0}(k,s))\exp(-j\frac{4\pi }{c}(f_{0}+\beta \hat{t})vt_{n}\cos\theta_{k})\\ & \cdot \exp(-j\frac{2\pi }{c}(f_{0}+\beta \hat{t})dm\sin\theta_{k}) \end{array}$$

In this expression, the first exponential term corresponds to range information, the second term contains velocity and angle information and indicates the coupling between range and velocity, while the third exponential term corresponds to angle information and reflects the coupling between range and array element position. In most practical scenarios, the radar-to-target distance is much larger than the array aperture, and therefore, the influence of array element position on the range term can be ignored. By performing a time-frequency substitution on the fast time $\hat{t}$, i.e., $\hat{b}\hat{t}\;\to \;f_{r}$, and applying the Keystone transform to remove the range-velocity coupling, a scaling operation is performed on the slow time $t_{n}$, such that $t_{n}=\frac{f_{0}}{f_{0}+f_{r}}t_{n}^{\prime }$, the expression in (16) becomes

(17) $$\begin{array}{ll} S_{beat}^{\prime }(f_{r},n,m) & =\sum_{k=1}^{K}\exp(j\frac{4\pi }{c}(f_{0}+f_{r})R_{k}(k,s))\exp(-j\frac{4\pi }{c}f_{0}vt_{n}^{\prime }\cos\theta_{k})\\ & \cdot \exp(-j\frac{2\pi }{c}f_{0}dm\sin\theta_{k}) \end{array}$$

It can be seen that the Keystone transform eliminates the coupling between range and velocity and compensates for the range walk of the target.

## 3. Proposed Method

This section presents a forward-looking FMCW radar imaging method under sparse sampling intervals. First, echo data are collected at different platform positions to construct a sparse slow-time sampling model. Then, the characteristics of false targets introduced by sparse sampling are analyzed. Subsequently, an adaptive beamforming-based algorithm is proposed to suppress these false targets. Finally, the proposed method is further analyzed and discussed.

### 3.1. Echo Signal Model Under Sparse Sampling Intervals

In the sparse sampling model proposed in this paper, the radar acquires measurements at a sampling interval $T_{s}$ where $T_{s}=iT$, and *i* is the interval factor. When i>1, the sampling becomes sparse. Based on (17), the sparse slow-time sampling yields the following range-angle vector model for the *m*-th receive element:

(18) $$X_{m}(\theta ,R)=A(\theta )S(k)B(R)+\varepsilon$$

where A(θ) is the slow-time steering matrix in the angle domain, given in (19); S(k) is the signal matrix, defined in (20); B(R) is the range steering matrix, expressed in (21); and ε denotes additive white Gaussian noise.

(19) $$A(\theta )={\left[a(\theta_{1})\;a(\theta_{2})\;\cdots \;a(\theta_{K})\right]}_{N\times K}$$

(20) $$S(k)=diag{\left[s_{1}\;\;\;\;s_{2}\;\;\;\cdots \;s_{K}\right]}_{K\times K}$$

(21) $$B(R)={\left[b(R_{1})\;b(R_{2})\;\cdots \;b(R_{K})\right]}_{K\times L}^{T}$$

where

(22) $$\begin{array}{ll} a(\theta_{k}) & =[1\;\;\exp(-j4\pi \frac{(f_{0}+f_{r})ivT\cos\theta_{k}}{\lambda f_{0}})\;\;\;\\ & \exp(-j4\pi \frac{(f_{0}+f_{r})2ivT\cos\theta_{k}}{\lambda f_{0}})\;\;\\ & \cdots \exp(-j4\pi \frac{(f_{0}+f_{r})(N-1)ivT\cos\theta_{k}}{\lambda f_{0}}){]}^{T} \end{array}$$

(23) $$s_{k}=\exp(-j2\pi dm\sin\theta_{k}/\lambda )\exp(j4\pi R_{0}(k,s)/\lambda )$$

(24) $$b(R_{k})=\exp(j4\pi f_{r}R_{0}(k,s)/c)$$

where T denotes the matrix transpose, diag(·) represents a diagonal matrix, and *L* is the number of fast-time samples. To reduce computational complexity, the two-dimensional range-angle estimation is decomposed into two one-dimensional procedures: an FFT is applied along the range dimension to obtain the range profile, and angle estimation is then performed on the slow-time data X corresponding to the range bins containing targets. The covariance matrix of X is

(25) $$R_{X}=E\left[XX^{H}\right]\approx \frac{1}{L}\sum_{1}^{L}XX^{H}$$

where H denotes the Hermitian (conjugate) transpose. The output power of the CBF at this stage is given by

(26) $$P_{CBF}=a^{H}(\theta )R_{X}a(\theta )$$

When the mainlobe beamwidth is smaller than the angular separation between two targets, they can be resolved in the angle domain. However, sparse sampling introduces grating lobes, whose effects will be analyzed in detail in the next subsection.

### 3.2. Doppler Ambiguity and Grating-Lobe Analysis

After obtaining the target range information via FFT along the range dimension, the physical array is used to perform coarse angle estimation. Based on the estimated angle, the locations of grating lobes and ambiguous targets can be computed.

Due to the use of sparse sampling, grating lobes arise in the angle domain. The angular positions of these grating lobes are given by

(27) $$\theta_{g}=\arccos(\cos\theta \pm \frac{q\lambda f_{0}}{2(f_{0}+f_{r})ivT}),q=1,2,\cdots$$

where θ is the true angle of the target, λ is the wavelength, and v is the speed of the platform movement. For narrowband signals, the condition $f_{0}\gg f_{r}$ holds. Therefore, (27) can be approximated as

(28) $$\theta_{g}=\arccos(\cos\theta \pm \frac{q\lambda }{2ivT}),q=1,2,\cdots$$

**Figure 2:**
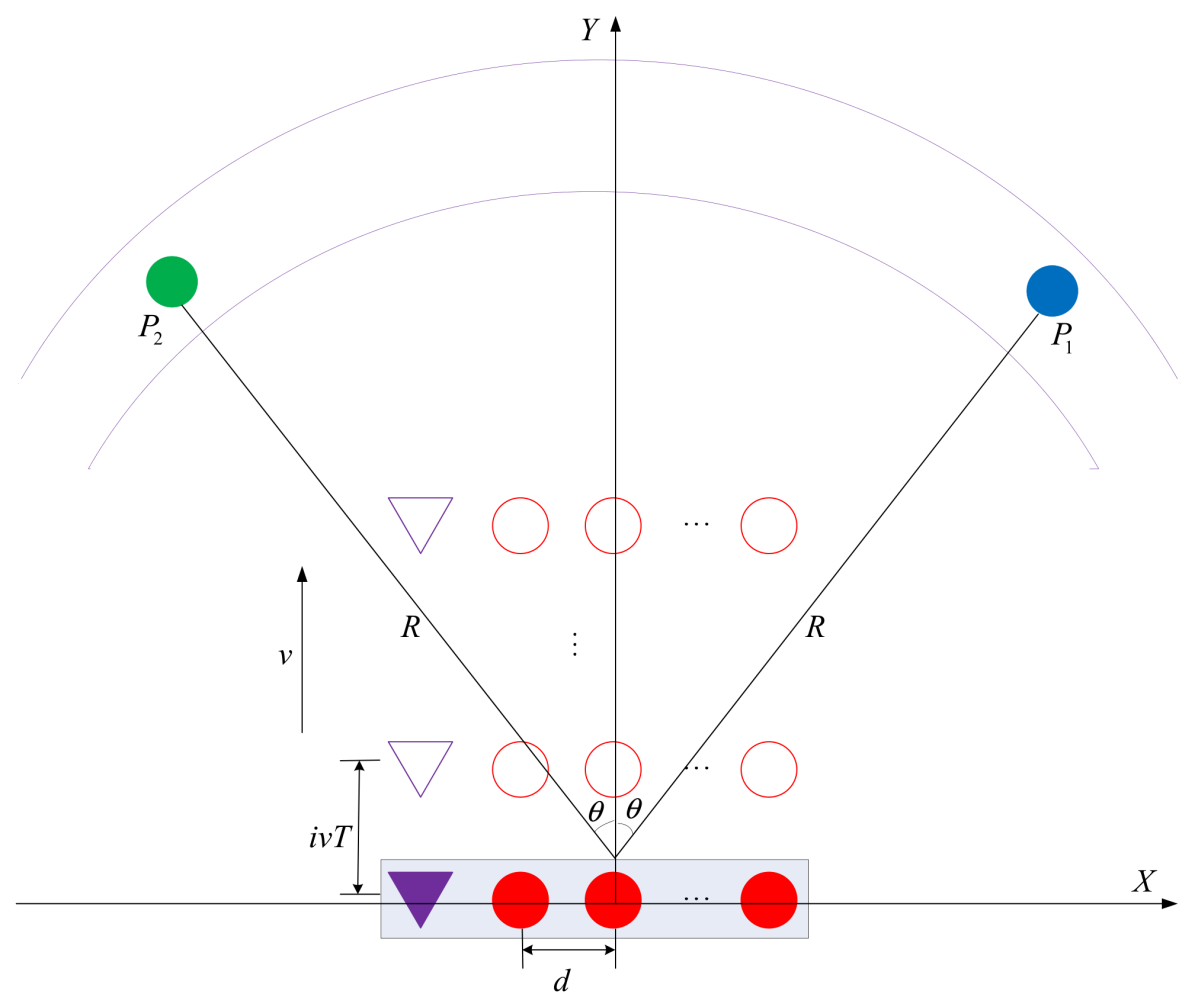
Schematic diagram of symmetrical target.

In the scenario illustrated in Figure 2, there exist two point targets symmetric with respect to the platform trajectory, denoted by $P_{1}(R\sin\theta ,R\cos\theta )$ and $P_{2}(-R\sin\theta ,R\cos\theta )$, where $R=R_{0}\left(1,m\right)=R_{0}\left(2,m\right)$ is the distance between the *m*-th receive element and the targets, and θ is the absolute value of the target angle. According to the geometric relationships, their instantaneous slant ranges at the receiver are given by

(29) $$R_{r,1}(t,m)=\sqrt{R^{2}+{\left(vt\right)}^{2}-2Rvt\cos\theta }-dm\sin\theta$$

(30) $$R_{r,2}(t,m)=\sqrt{R^{2}+{\left(vt\right)}^{2}-2Rvt\cos\theta }+dm\sin\theta$$

The instantaneous slant ranges from the transmitter are

(31) $$R_{s,1}(t)=\sqrt{R_{0}^{2}\left(1,s\right)+{\left(vt\right)}^{2}-2R_{0}\left(1,s\right)vt\cos\theta }$$

(32) $$R_{s,2}(t)=\sqrt{R_{0}^{2}\left(2,s\right)+{\left(vt\right)}^{2}-2R_{0}\left(2,s\right)vt\cos\theta }$$

where $R_{0}\left(1,s\right)$ and $R_{0}\left(2,s\right)$ denote the initial ranges from the transmit element to the two targets, respectively. It can be seen that the two targets have the same transmit slant range, while their receive slant ranges differ by

(33) $$\Delta R_{1,2}=R_{r,2}(t,m)-R_{r,1}(t,m)=2dm\sin\theta \ll \rho_{r}$$

Equation (33) shows that the instantaneous slant-range difference between the two targets is much smaller than the range resolution. Therefore, the two symmetric point targets have essentially identical instantaneous slant ranges and, consequently, identical Doppler histories. After angle estimation, this results in false targets that appear symmetrically about the zero-angle axis. In addition, the grating lobes described in (28) also generate false targets symmetric with respect to the zero-angle axis. Hence, the final angular positions of the false targets are given by

(34) $$\theta_{g}=\pm \arccos(\cos\theta \pm \frac{q\lambda }{2ivT}),q=1,2,\cdots$$

### 3.3. False-Target Suppression Algorithm Based on Adaptive Beamforming

To suppress the angular-domain false targets that arise in forward-looking imaging, this subsection proposes a false-target suppression algorithm based on adaptive beamforming.

Assume that a target $P_{k1}\left(R_{k}sin\theta_{k},R_{k}\cos\theta_{k}\right)$ exists on one side of the radar platform’s trajectory. After receiving the target echo, an FFT is applied along the fast-time dimension to achieve range focusing. The range-focused result at the *m*-th receive element is given by

(35) $$\begin{array}{ll} f(f_{r},m) & =\sin c(\pi T(f_{r}+\frac{2\beta R_{k}}{c}))\exp(j\frac{4\pi }{\lambda }R_{k})\\ & \;\;\;\;\;\;\cdot \exp(-j\frac{2\pi }{\lambda }dm\sin\theta ) \end{array}$$

For the range bin corresponding to target $P_{k1}$, CBF is applied to the slow-time data to obtain an angle estimate, yielding the range-angle response $f(m,R_{k},\theta_{k})$ for the m-th receive channel. Based on the previous analysis, the resulting range-angle map contains not only the true target $P_{k1}$ but also the ambiguous target $P_{k2}$ and the grating-lobe-induced false targets $P_{g,i}$. Therefore, the range-angle estimate can be expressed as

(36) $$\begin{array}{l} f_{P_{k1}}(m,R,\theta )\\ =f(m,R_{k},\theta_{k})+f(m,R_{k},-\theta_{k})+\sum_{i=1}^{I}\left[f(m,R_{k},\theta_{g,i})+f(m,R_{k},-\theta_{g,i})\right]\\ =[U_{\theta_{k}}\;U_{-\theta_{k}}\;U_{\theta_{g,1}}\;U_{-\theta_{g,1}}\cdots U_{\theta_{g,I}}\;U_{-\theta_{g,I}}]\cdot \left[\begin{array}{l} \exp(-j2\pi dm\sin\theta_{k}/\lambda )\\ \exp(-j2\pi dm\sin(-\theta_{k})/\lambda )\\ \exp(-j2\pi dm\sin\theta_{g,1}/\lambda )\\ \exp(-j2\pi dm\sin(-\theta_{g,1})/\lambda )\\ \vdots \\ \exp(-j2\pi dm\sin\theta_{g,I}/\lambda )\\ \exp(-j2\pi dm\sin(-\theta_{g,I})/\lambda ) \end{array}\right] \end{array}$$

where I denotes the number of grating lobes on one side of the platform trajectory, and $U=[U_{\theta_{k}}\;U_{-\theta_{k}}\;U_{\theta_{g,1}}\;U_{-\theta_{g,1}}\cdots U_{\theta_{g,I}}\;U_{-\theta_{g,I}}]$ represents the complex scattering coefficients of the targets. By coherently summing the range-angle responses across all channels, the imaging result for the M receive elements can be written as

(37) $$\begin{array}{ll} F(R,\theta ) & =\sum_{m=1}^{M}f_{P_{k1}}(m,R,\theta )=U\cdot \bar{A}\\ & =U\cdot {\left[a(\theta_{k})\;a(-\theta_{k})\;a(\theta_{g,1})\;\cdots \;a(\theta_{g,I})\right]}^{T} \end{array}$$

where $\bar{A}$ is the array manifold that includes both the ambiguous target and the grating-lobe components, and

(38) $$a(\theta )={\left[1\;\exp(-j2\pi d\sin\theta /\lambda )\;\cdots \exp(-j2\pi (M-1)d\sin\theta /\lambda )\right]}^{T}$$

Using the degrees of freedom provided by the physical array, an adaptive spatial beam is formed based on the LCMV criterion, such that nulls are placed at the angular positions of the false targets. The cost function of the LCMV beamform

(39) $$\underset{w}{min\;}w^{H}R_{XX}w\;subject\;to\;{\bar{A}}^{H}w=f$$

where $R_{XX}$ is the autocorrelation matrix of the received signal, and f is the constraint response vector. By solving the above constrained optimization problem using the Lagrange multiplier method, the LCMV weight vector is obtained as

(40) $$w=R_{XX}^{-1}\bar{A}{\left({\bar{A}}^{H}R_{XX}^{-1}\bar{A}\right)}^{-1}f$$

By applying the weight vector to the imaging results of all channels, the final image with Doppler ambiguity resolved and grating lobes suppressed is given by

(41) $$\bar{F}(R,\theta )=w^{H}\cdot \bar{A}\cdot F(R,\theta )$$

### 3.4. Algorithm Procedure and Analysis

To address the large echo data volume and Doppler ambiguity present in forward-looking FMCW radar imaging, a false-target suppression algorithm under sparse sampling intervals is proposed. The overall algorithm procedure is summarized in Algorithm 1.
**Algorithm 1.** False-Target Suppression Under Sparse Sampling Intervals.1: Acquire sparsely sampled echo data through platform motion. 2: Perform range-shift compensation, residual video phase removal, and range walk correction. 3: Construct the sparse-sampling-based range-angle echo signal model. 4: Apply FFT along the range dimension and CBF along the angle dimension for each channel to obtain the initial range-angle image (containing grating lobes and ambiguous targets). 5: For targets in different range bins, use the physical array to obtain coarse angle estimates and compute the angles of grating-lobe and ambiguous targets. 6: Apply adaptive beamforming to place nulls at the false-target angles, and coherently combine the weighted outputs across channels to obtain an ambiguity-free and grating-lobe-free range-angle image.

In this subsection, we analyze the computational complexity of the proposed method. The overall complexity primarily arises from three components:

(i) Time-domain FFT processing;
(ii) Spatial-domain CBF processing;
(iii) The weighting operation for suppressing false targets.

The total complexity is given by

(42) $$CC_{\mathrm{total}}=CC_{\mathrm{time}}+CC_{\mathrm{space}}+CC_{\mathrm{weighting}}$$

Here, $CC_{\mathrm{time}}$ denotes the computational complexity of time-domain processing,$CC_{\mathrm{space}}$ corresponds to spatial-domain processing, and $CC_{\mathrm{weighting}}$ represents the complexity of the weighting operation, expressed as

(43) $$CC_{\mathrm{time}}=O(MNL\log L+2MLN^{2})$$

(44) $$CC_{\mathrm{space}}=O(2M^{2}(K_{\mathrm{true}}+K_{\mathrm{false}})+2M{\left(K_{\mathrm{true}}+K_{\mathrm{false}}\right)}^{2}+M(K_{\mathrm{true}}+K_{\mathrm{false}}))$$

(45) $$CC_{\mathrm{weighting}}=O(1200LM)$$

where $K_{\mathrm{true}}$ is the number of true targets, $K_{\mathrm{false}}$ is the number of false targets, and the constant 1200 is determined by the angular search range (−60°~60°).

The method in [31] analyzes left-right Doppler ambiguity in imaging and proposes a high-resolution adaptive beamforming approach with spatial null constraints. Under the same angular resolution, its computational complexity is

(46) $${\bar{CC}}_{\mathrm{total}}={\bar{CC}}_{\mathrm{time}}+{\bar{CC}}_{\mathrm{space}}+{\bar{CC}}_{\mathrm{weighting}}$$

where

(47) $${\bar{CC}}_{\mathrm{time}}=O(iMNL\log L+2iMLN^{2})$$

(48) $$\begin{array}{ll} {\bar{CC}}_{\mathrm{space}} & =O(2M^{2}(K_{true}+K_{false})+\\ & 2M{\left(K_{true}+K_{false}\right)}^{2}+M(K_{true}+K_{false})) \end{array}$$

(49) $${\bar{CC}}_{\mathrm{weighting}}=O(1200LM)$$

Comparing $CC_{\mathrm{time}}$ and ${\bar{CC}}_{\mathrm{time}}$, the proposed algorithm exhibits significantly lower computational complexity due to the adoption of sparse interval sampling, and this advantage becomes more pronounced as the interval factor increases. Comparing $CC_{\mathrm{space}}$ and ${\bar{CC}}_{\mathrm{space}}$, although the method in [31] generates fewer false targets (only ambiguous targets), their impact is negligible compared with the time-domain processing complexity. Overall, the proposed method offers a substantial computational advantage.

It should be noted that when the number of false targets becomes large, the effectiveness of null steering is limited by the degrees of freedom of the physical array. However, in most scenarios—especially when the range resolution is sufficiently high—each range bin typically contains only a small number of targets with different angles, allowing false targets to be suppressed on a per-range-bin basis. Additionally, the number of grating lobes can be reduced by properly designing the sparse sampling interval or the angular search range, or by employing MIMO radar to increase the available degrees of freedom.

In practice, the applicability of the proposed method should be clearly delineated against other existing techniques, namely compressed sensing (CS), Doppler beam sharpening (DBS), and conventional spatial adaptive processing, to provide useful engineering guidelines.

First, compared with CS-based methods, our approach is particularly advantageous under deterministic uniform sparse sampling when the platform motion is relatively steady and targets do not undergo severe maneuvers during the observation interval. In such quasi-stationary scenarios, the grating-lobe positions can be precisely predicted, which allows us to adopt a non-iterative FFT-plus-LCMV framework that is roughly two to three orders of magnitude faster than iterative CS reconstruction. This makes our method highly suitable for real-time automotive and UAV applications with limited power and computing budgets. However, if the SNR falls below approximately –10 dB or the target density exceeds the available array degrees of freedom, CS-based sparse recovery tends to be more robust, and our method may become less competitive.

Second, compared with DBS, which relies solely on single-channel Doppler histories and does not exploit spatial phase information, our method requires a well-calibrated multi-channel array to be available on the platform. When calibration is satisfactory, we jointly utilize temporal and spatial information to completely eliminate left–right ambiguity while achieving significantly higher angular resolution than DBS. Conversely, if the array suffers from severe unknown channel gain/phase mismatches (e.g., uncalibrated errors), DBS, whose performance is largely independent of array calibration, becomes more robust and would be a safer practical choice under such hardware-limited conditions.

Third, compared with conventional spatial adaptive processing (e.g., standard CBF/DBF): Our LCMV-based beamformer requires accurate estimation of the covariance matrix to place precise nulls. This is feasible when the number of available snapshots is sufficient and the false-target angles are known (which we can predict theoretically). Under these conditions, our method achieves significantly superior angular resolution compared with traditional CBF while effectively suppressing false targets. However, in scenarios with a very limited number of snapshots or rapid dynamic changes, employing a simple diagonal-loaded DBF may offer better stability; thus, this simplified approach is more recommended for such cases.

## 4. Simulation and Experimental Results

To demonstrate the effectiveness of the proposed method, both simulation and experimental studies are carried out in this section. It is worth noting that this work focuses on achieving ambiguity-free and grating-lobe-free range-angle imaging with a limited number of samples; the blind-zone problem inherent to forward-looking radar is not considered here.

### 4.1. Simulation

A simulated single-transmit multiple-receive uniform linear array radar is used to validate the proposed algorithm. The simulation scenario satisfies the far-field and narrowband assumptions. The detailed simulation parameters are listed in Table 1.

The radar moves at a constant velocity along the array’s normal direction and samples the echo signals at different platform positions. The interval factor is set to 4, and a total of 25 samples are collected. A point target is located in the forward-looking region, with an initial range of 20 m and an angle of 45°.

**Figure 3:**
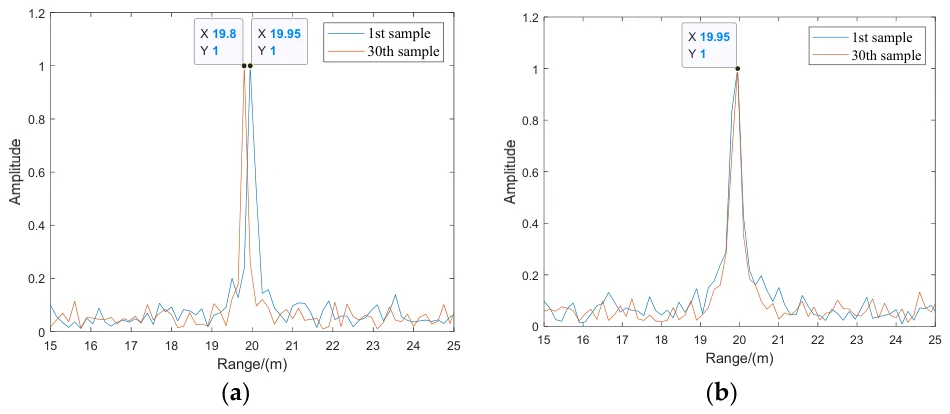
Results of range migration correction. (**a**) Before range migration correction. (**b**) After range migration correction.

Figure 3 illustrates the target range before and after range walk correction. In Figure 3a, the target ranges corresponding to the first and thirtieth samples are 19.80 m and 19.95 m, respectively. The range walk exceeds one range resolution cell, indicating that the error cannot be ignored. After applying the range walk correction, the result shown in Figure 3b gives a stabilized target range of 19.95 m, demonstrating the effectiveness of the proposed algorithm.

**Figure 4:**
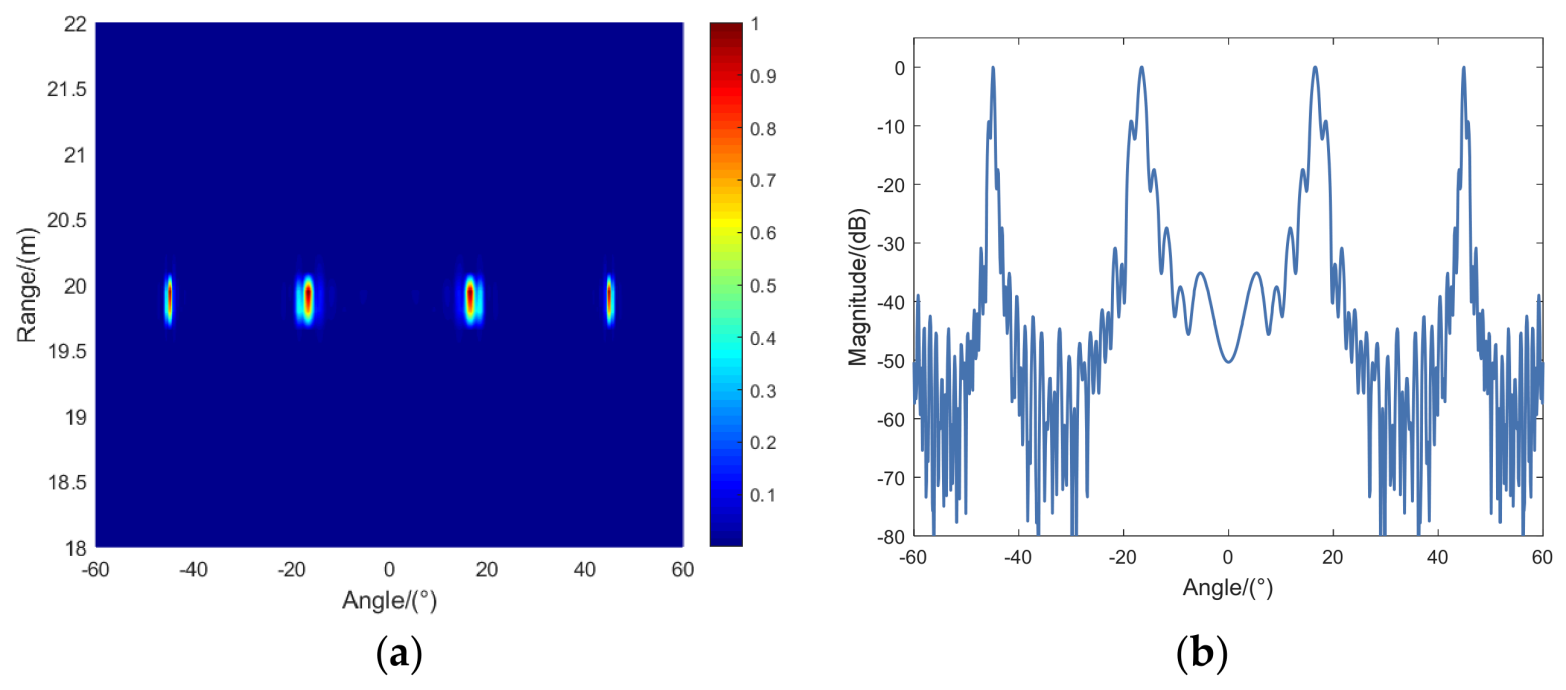
Imaging result before algorithm processing. (**a**) Untreated range-angle image. (**b**) Angle dimension slice before algorithm processing.

Figure 4a shows the unprocessed range-angle image, where multiple false targets are clearly observed in the angle domain. The angular slice in Figure 4b reveals four peaks located at −44.9°,−16.5°,16.5° and 44.9°. Among these, only the peak at 44.9° corresponds to the true target, while the others are false targets, which is consistent with the theoretical analysis.

**Figure 5:**
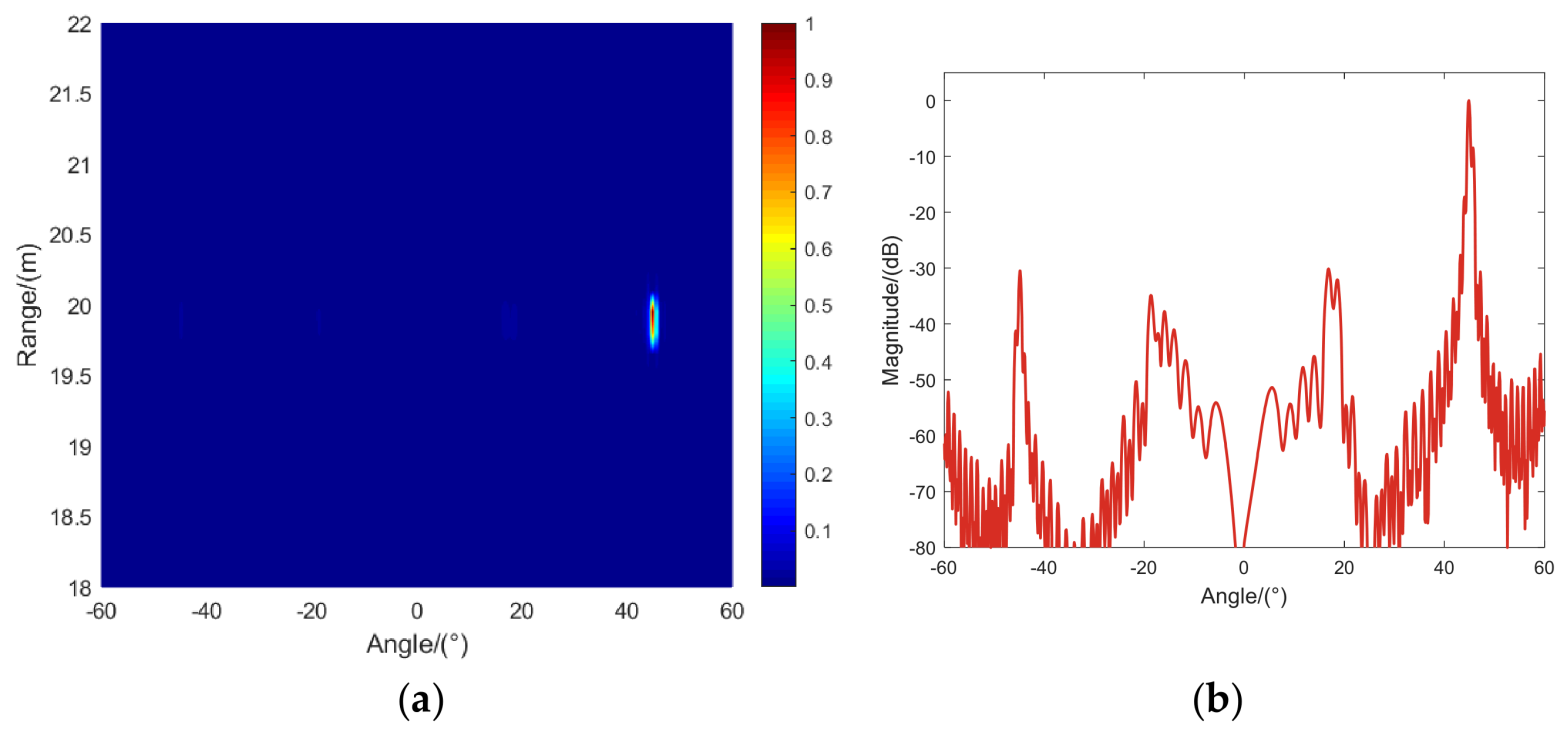
Imaging result after algorithm processing. (**a**) Range-angle image after algorithm processing. (**b**) Angle dimension slice after algorithm processing.

Figure 5 presents the imaging result after applying the proposed algorithm. All false targets are suppressed by more than 30 dB, while the true target is preserved, effectively eliminating both Doppler ambiguity and grating-lobe artifacts.

**Figure 6:**
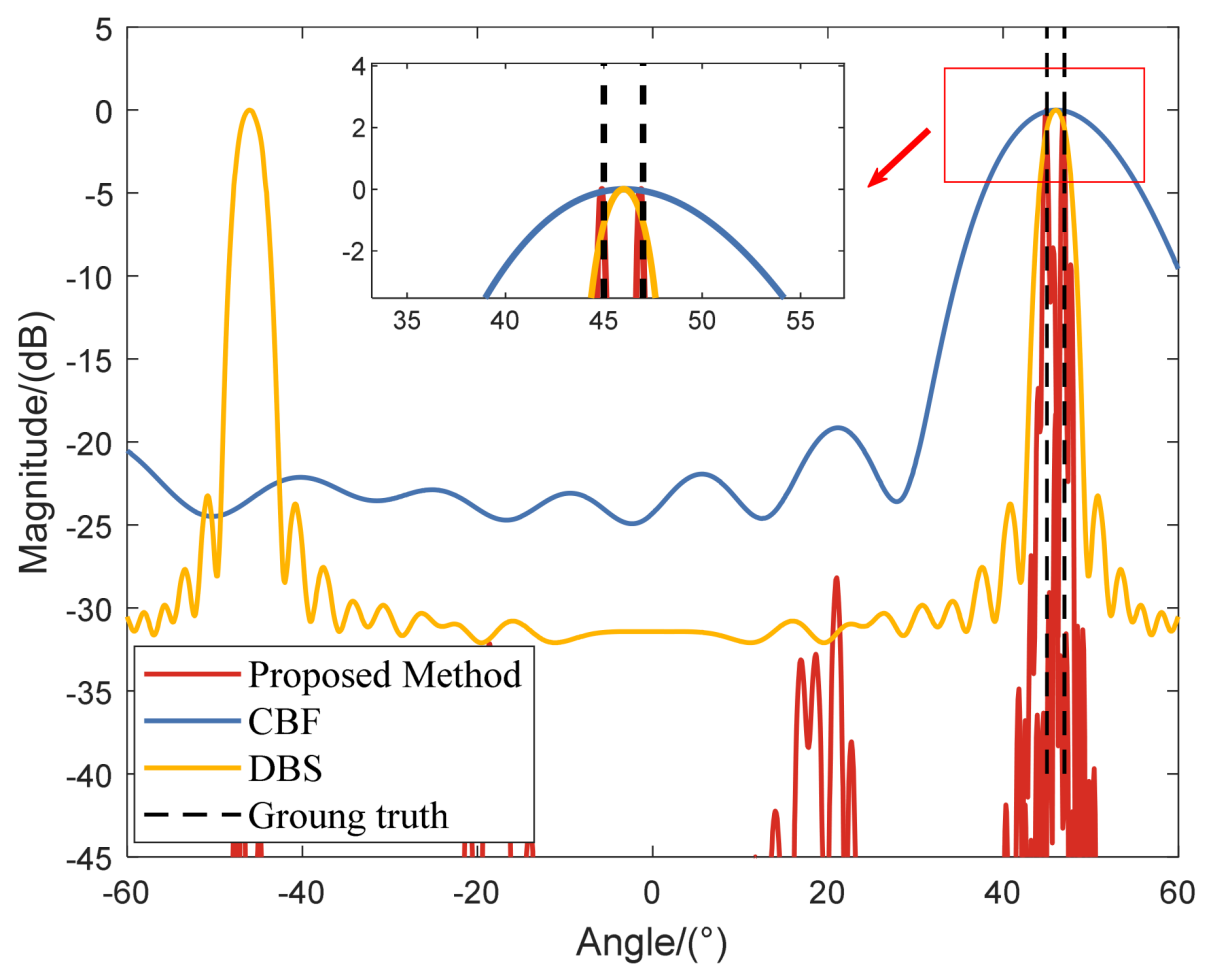
Comparison of different algorithms for targets at 45° and 47°.

**Figure 7:**
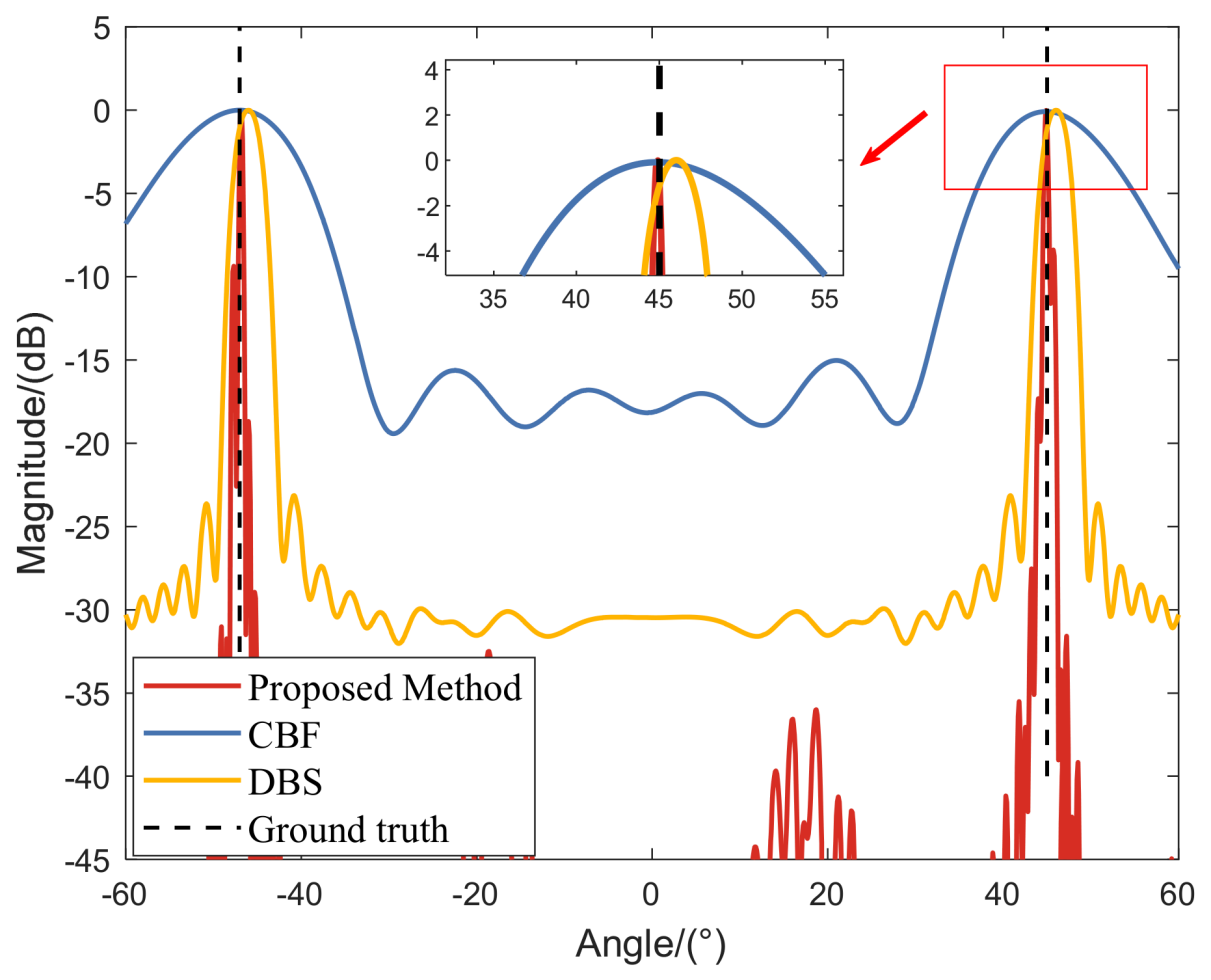
Comparison of different algorithms for targets at 45° and −47°.

**Figure 8:**
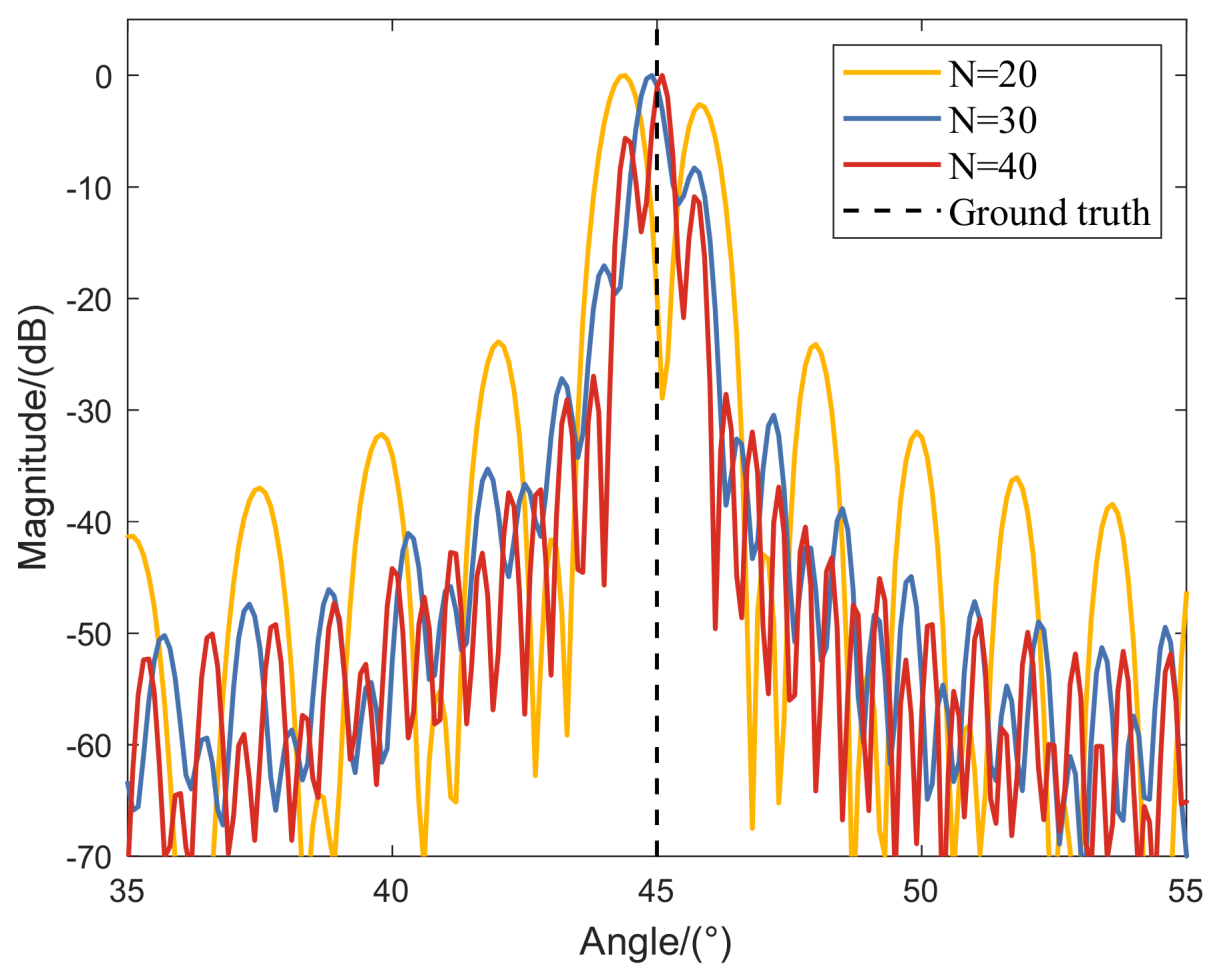
Performance under different numbers of samples.

To evaluate the algorithm’s performance in multi-target scenarios and compare it with the conventional CBF [9] and DBS [28] methods under continuous sampling mode with the same aperture, a series of simulations are conducted, with results shown in Figure 6, Figure 7 and Figure 8. In these comparisons, the CBF algorithm employs an 8-element physical array, while the DBS algorithm uses the same number of continuous samples as the proposed method (30 samples).

Assume two targets located at 45° and 47°. The results are shown in Figure 6. The proposed algorithm effectively suppresses false targets, and the estimated angles are 44.9° and 46.9°, indicating that the two targets are clearly distinguishable. In contrast, the CBF algorithm exhibits insufficient resolution and fails to separate the two targets. Although the DBS algorithm offers better resolution than CBF, it still cannot resolve the two closely spaced targets and suffers from Doppler ambiguity.

To demonstrate the effectiveness of the algorithm when targets are located on both sides of the radar trajectory, a simulation is conducted with two point targets positioned at 45° and −47°. The results, shown in Figure 7, indicate that the proposed algorithm estimates the target angles as 44.9° and −46.9°, respectively, confirming its effectiveness in this scenario.

Figure 8 evaluates the proposed algorithm under different numbers of samples. As the number of samples increases, the mainlobe becomes narrower and the sidelobes decrease, indicating that the angular resolution improves with an increasing number of sampling positions.

**Figure 9:**
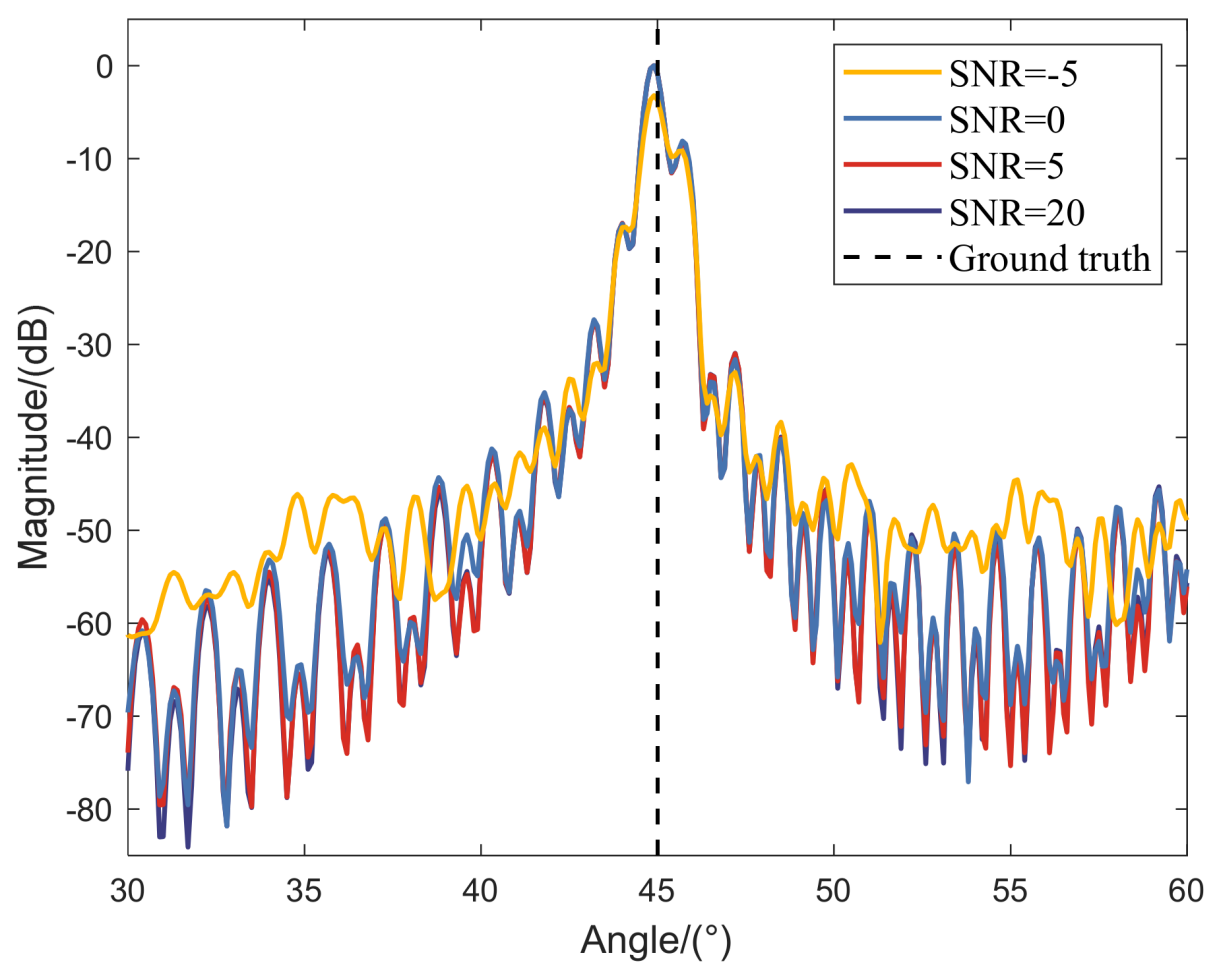
Performance under different SNR levels.

Figure 9 presents the performance of the proposed algorithm under different SNR conditions. The simulation uses the same parameters as listed in Table 1, with a sampling interval factor of *i* = 4 and a total number of samples *N* = 25. The results show that the algorithm remains effective even at a low SNR of −5 dB, demonstrating strong robustness to noise.

**Figure 10:**
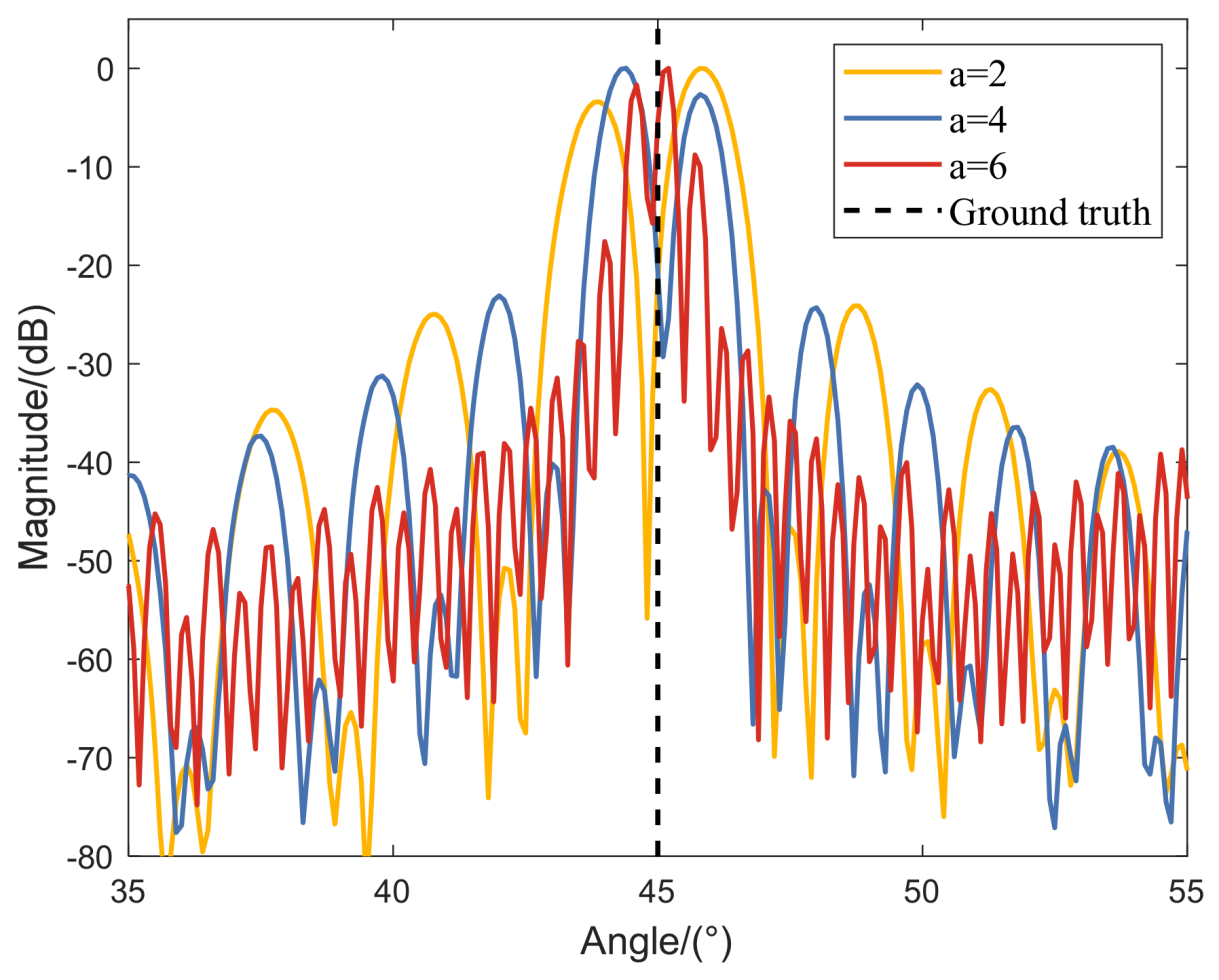
Performance under different sampling intervals.

As shown in Figure 10, the proposed algorithm is evaluated under different sampling intervals while keeping the total number of samples fixed (*N* = 30). The results indicate that as the sampling interval increases, the mainlobe becomes progressively narrower and the sidelobes diminish. This demonstrates that the angular resolution improves with larger sampling intervals.

### 4.2. Experiments

**Figure 11:**
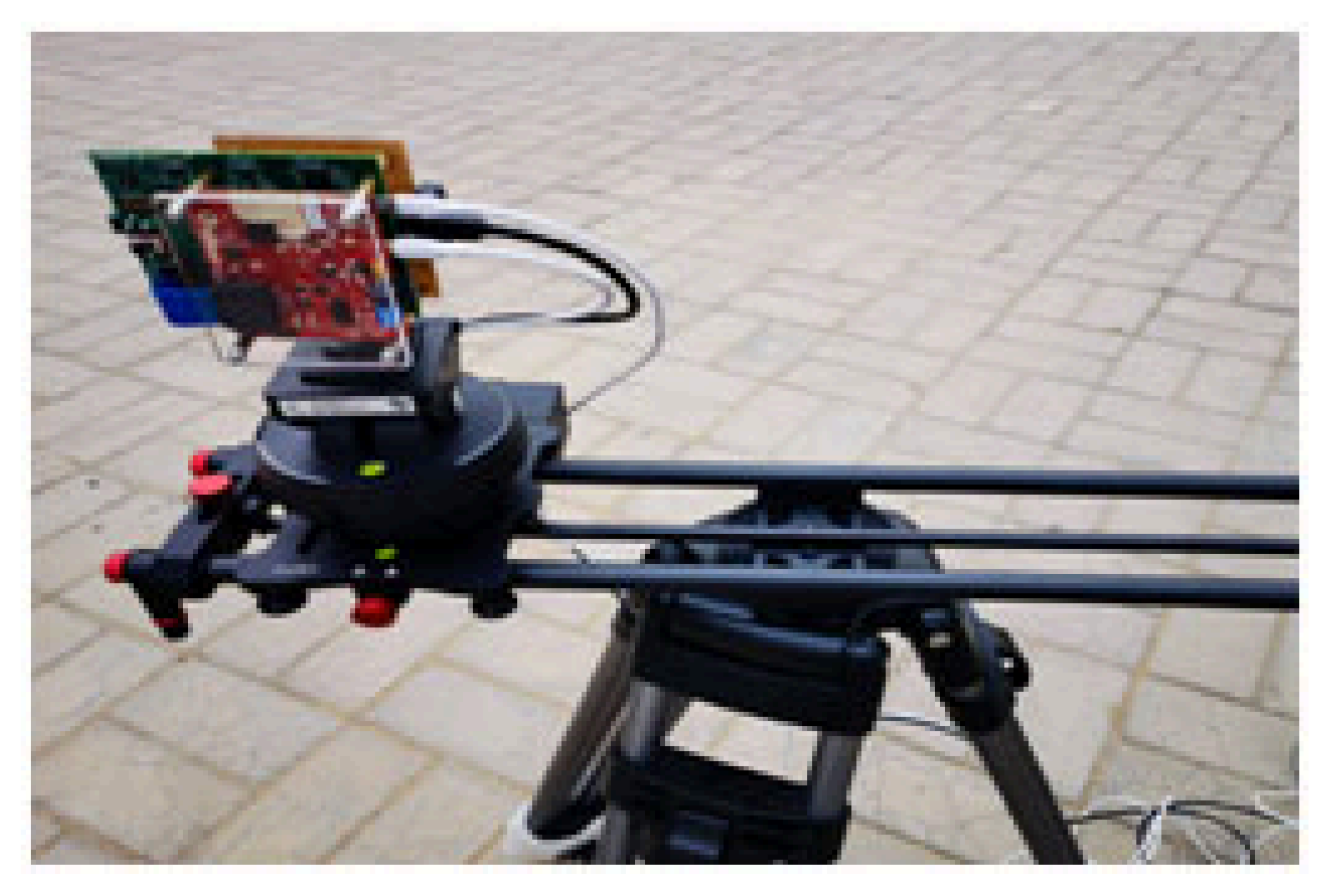
Linear slider and radar setup.

The TI IWR1443 radar is used to validate the effectiveness of the proposed algorithm. The radar is mounted on a linear slider, as shown in Figure 11, and the experimental parameters are listed in Table 2.

**Figure 12:**
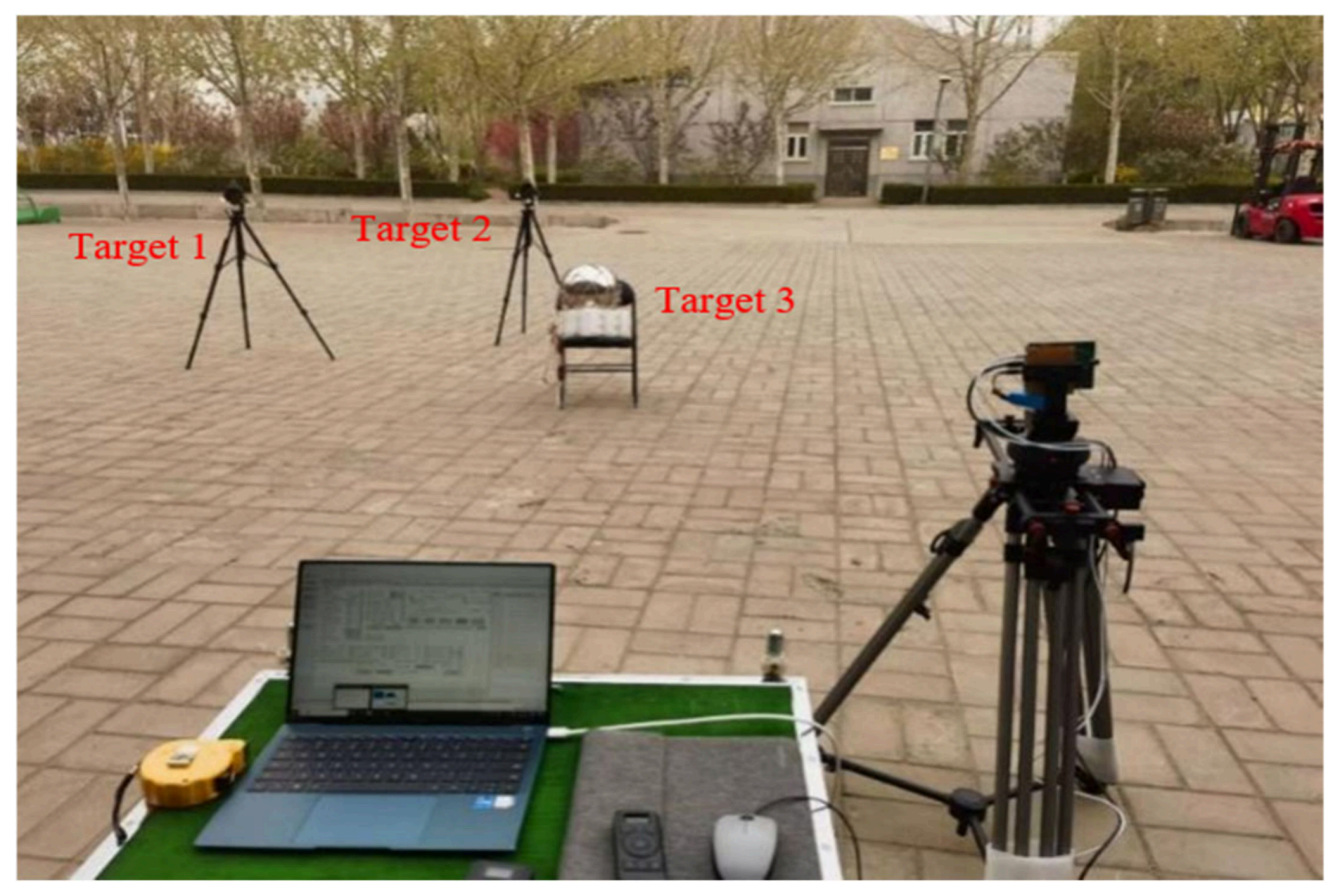
Experimental scenario A.

**Experiment A**: During the experiment, the radar moves at a constant velocity along the slider, and three targets (two corner reflectors and a metallic sphere) are placed within the forward-looking region. The target parameters are provided in Table 3, and the experimental setup is illustrated in Figure 12.

**Figure 13:**
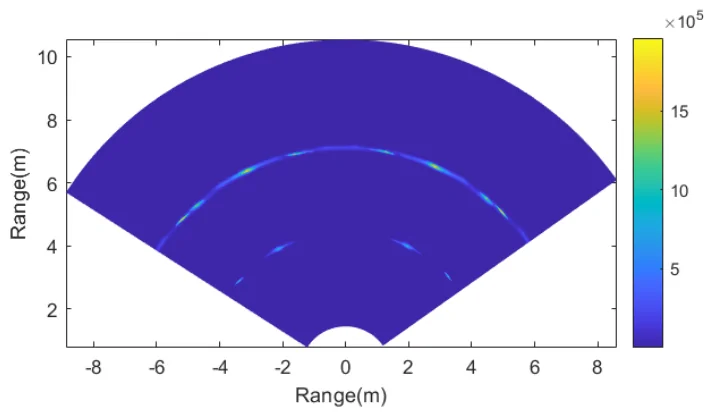
Imaging result before false-target suppression (Experiment A).

**Figure 14:**
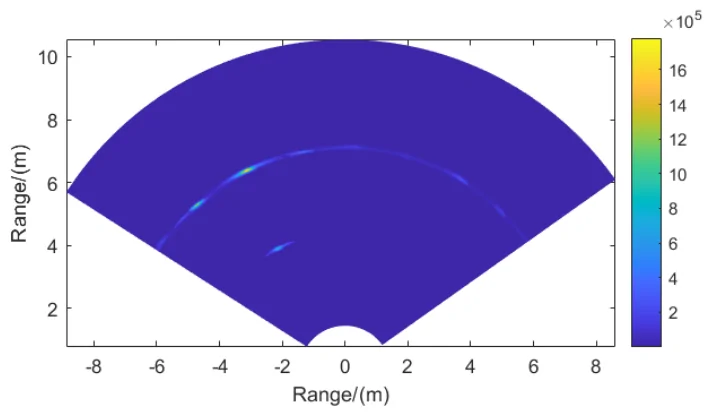
Imaging result after false-target suppression (Experiment A).

The experimental results are shown as follows. Figure 13 presents the imaging result before false-target suppression, where real targets, grating lobes, and ambiguous targets are all observed. After applying the proposed algorithm, these false targets are effectively suppressed, as illustrated in Figure 14. These results verify the effectiveness of the proposed method in practical scenarios.

**Figure 15:**
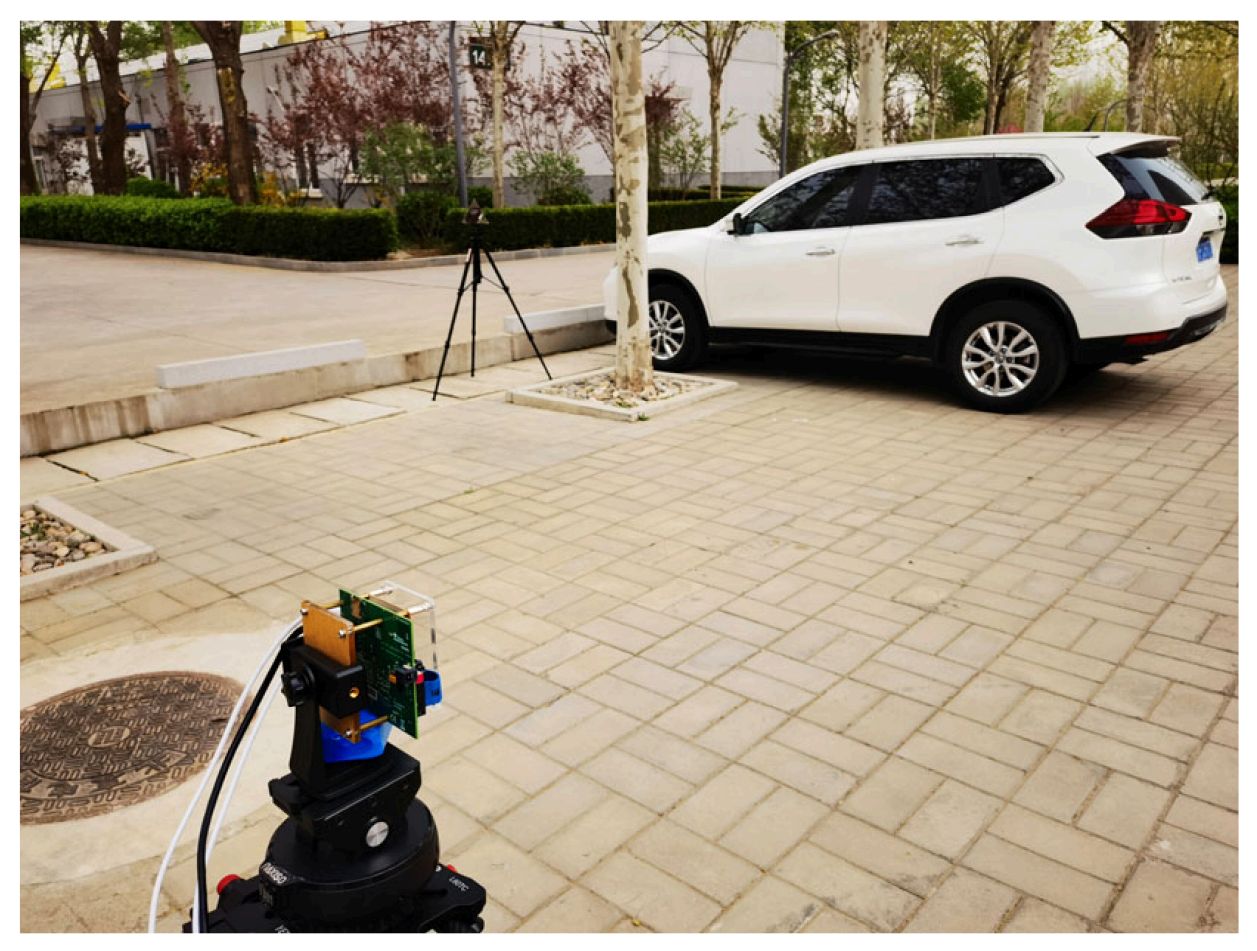
Experimental scenario B.

**Experiment B**: In this experiment, two targets are considered: a corner reflector (point target) and a vehicle (extended target). The vehicle has a range extent from approximately 6 m to 7.8 m, with an angular extent from approximately –35° to –15°. The corner reflector is located at a range of approximately 7.2 m and an angle of approximately –47°. The experimental setup is illustrated in Figure 15.

**Figure 16:**
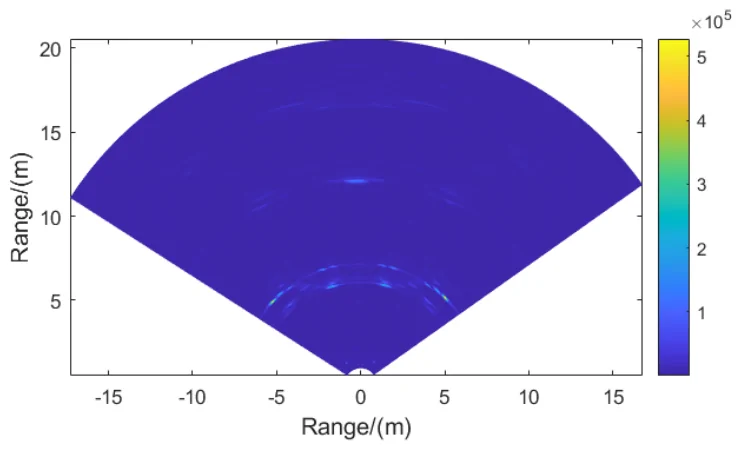
Imaging result before false-target suppression (Experiment B).

**Figure 17:**
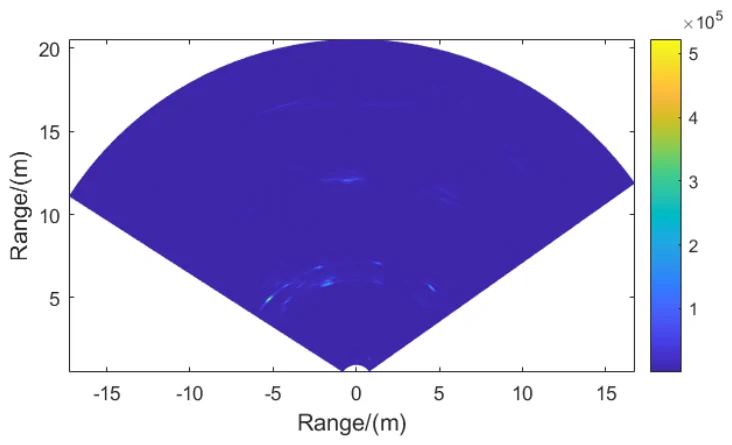
Imaging result after false-target suppression (Experiment B).

The experimental results are shown below. Figure 16 presents the imaging result before false-target suppression, which includes the true targets (the corner reflector and multiple scattering points from the vehicle), as well as grating lobes and ambiguous targets. After applying the proposed algorithm, the false targets are effectively suppressed, as shown in Figure 17. These experimental results verify the effectiveness of the proposed algorithm in scenarios involving both point targets and extended targets.

## 5. Conclusions

This paper addresses several key challenges in forward-looking imaging for small platforms such as vehicles and unmanned aerial vehicles, including limited angular resolution, real-time processing constraints, and Doppler ambiguity. A forward-looking FMCW radar imaging method based on sparse sampling intervals is proposed. By employing sparse platform motion to synthesize a large virtual aperture, the method enhances angular resolution despite the limitation of the physical array aperture. Compared with traditional SAR and DBS approaches, the proposed method significantly reduces the required echo data volume, thereby lowering the computational burden on the platform. Furthermore, adaptive beamforming is applied to suppress false targets, enabling the reconstruction of high-resolution range-angle images free of Doppler ambiguity and grating lobes. Both simulation and experimental results verify the effectiveness of the proposed method.

Future work will focus on analyzing the impact of platform motion errors and corresponding compensation strategies in depth.

## Figures and Tables

**Table 1:** Radar parameters.

Parameter	Value
Carrier frequency $f_{0}$/(GHz)	77
Bandwidth B/(GHz)	1
Modulated period T/(us)	100
Number of elements M	8
Wavelength λ/(m)	0.004
Elements spacing d/(m)	0.002
Velocity v/(m/s)	20

**Table 2:** Experimental parameters.

Parameter	Value
Carrier Frequency /(GHz)	77
Bandwidth /(GHz)	3
Slope/(MHz/μs)	68
Modulated Period /(μs)	44
Sampling Frequency /(MHz)	11.6
Number of elements	4
Wavelength /(m)	0.004
Elements spacing /(m)	0.002
Platform Velocity /(m/s)	0.03
Sample Interval	4
Sampling Number	11

**Table 3:** Experimental target parameters.

Target	Type	Range/(m)	Angle/(deg)
Target1	Corner reflector	7	−45
Target2	Corner reflector	7.04	−21
Target3	Metallic sphere	4.5	−21

## Data Availability

The data used to support the findings of this study are available from the corresponding author upon reasonable request.
